# Mediating effects of lipid metabolic and inflammatory factors on skeletal muscle mass in adults: A cross-sectional study

**DOI:** 10.1097/MD.0000000000049428

**Published:** 2026-06-19

**Authors:** Jingran Hu, Bin Gu, Fei Wang

**Affiliations:** a Department of Rehabilitation, Korea Nazarene University, Cheonan, Republic of Korea; b China Rehabilitation Research Center, Beijing, China; c School of Physical Education, Shanxi University, Taiyuan, Shanxi, China; d Sports Science Institute, Shanxi University, Taiyuan, Shanxi, China.

**Keywords:** body mass index, inflammation, metabolism, skeletal muscle mass, structural equation modeling

## Abstract

Investigating the mediating effects of metabolic and inflammatory factors on skeletal muscle mass is essential, as these factors may help deepen understanding of the complex processes underlying skeletal muscle mass decline and support effective prevention and management in adults. Health examination data from 2277 participants who attended the health examination center of a provincial hospital in Taiyuan, China, between 2019 and 2023 were collected. Random forest analysis, correlation analysis, regression modeling, and mixed-effects structural equation modeling were used to identify determinants of appendicular skeletal muscle index (ASMI) and to examine metabolic and inflammatory mediating pathways. ASMI was significantly higher in obese participants than in nonobese participants (*P* < .001). Body mass index (BMI) was significantly positively correlated with ASMI (*r* = 0.68, *P* < .001) and negatively associated with the risk of low skeletal muscle mass (OR = 0.53, 95% CI: 0.47–0.60). Structural equation modeling showed that BMI not only had a direct positive effect on ASMI, but also influenced ASMI through metabolic and inflammatory pathways. In the high-BMI group, lipid metabolism had a direct positive effect on ASMI (*β* = 0.08, *P *< .001). In contrast, in the low-BMI group, lipid metabolism had a direct negative effect on ASMI (*β* = −0.08, *P* < .001) and was also positively associated with ASMI through inflammatory pathways (*β* = 0.07, *P* < .001). In this group, glucose metabolism (*β* = 0.24, *P* < .001) and protein metabolism (*β* = −0.08, *P* < .001) regulated lipid metabolism and indirectly influenced skeletal muscle mass through lipid metabolic pathways. BMI was significantly positively associated with ASMI, with its effects mainly mediated through lipid metabolism and inflammatory pathways, and the pathways differed across BMI groups. These findings highlight lipid metabolism and inflammation as key factors associated with muscle health, facilitating more accurate risk screening and the implementation of targeted prevention and management strategies.

## 1. Introduction

Sarcopenia is an age-related, progressive, and systemic skeletal muscle disorder characterized by generalized loss of muscle mass and reduced muscle strength reaching specific diagnostic thresholds, which increases the risk of adverse outcomes such as falls, disability, frailty, and mortality.^[1]^ The decline in skeletal muscle mass begins in middle age and accelerates with age, often considered an early sign and key indicator of sarcopenia, playing a crucial role in assessing the risk of abnormal body composition and metabolic dysfunction.^[2]^ The global prevalence of sarcopenia is estimated to range from 10 to 27%, with variations influenced by differences in age, ethnicity, and methods used for muscle mass assessment.^[3]^ Although it is traditionally considered a condition affecting older adults, increasing evidence suggests that decreased skeletal muscle mass is also observed among non-elderly populations, particularly individuals over 40 years of age.^[4]^ In addition, several studies have reported that some middle-aged individuals with obesity may simultaneously exhibit reduced skeletal muscle mass,^[5]^ suggesting that excessive adiposity and decreased skeletal muscle mass may coexist. This combined condition may confer a substantially greater risk to overall health.

With the continued escalation of the global obesity epidemic, the prevalence of overweight and obesity among Chinese adults has also increased. Currently, approximately half of the adult population in China is overweight or obese, and this proportion is projected to further increase to 65.3% by 2030.^[6]^ Obesity is typically accompanied by metabolic dysregulation and chronic low-grade inflammation, both of which are considered to play important roles in the decline of skeletal muscle mass.^[7]^ Meanwhile, skeletal muscle itself is also involved in glucose uptake, glycogen storage, and lipid metabolism; therefore, reduced skeletal muscle mass may further contribute to metabolic dysregulation and adverse health outcomes.^[8]^ Among metabolic disturbances, lipid metabolism disorders are characterized by elevated levels of total cholesterol (TC), triglycerides (TG), and low-density lipoprotein cholesterol (LDL-C), along with decreased levels of high-density lipoprotein cholesterol (HDL-C). These alterations may indirectly affect muscle structure and function by promoting fat accumulation, oxidative stress, and inflammatory responses.^[9]^ Conversely, as skeletal muscle serves as a major site for fatty acid oxidation, reductions in skeletal muscle mass may further aggravate lipid metabolism disorders, thereby forming a vicious cycle.^[10]^ A study conducted in Korea reported that low skeletal muscle mass index was significantly positively associated with the risk of dyslipidemia, with an approximately 23% increased risk after adjustment for potential confounding factors, and this association remained independent of abdominal obesity status.^[11]^

Similar to lipid abnormalities, glucose metabolism disorders are also prevalent among individuals with obesity, typically manifested as elevated levels of glycated hemoglobin (HbA1c) and insulin (INS).^[12]^ Previous studies have demonstrated a significant association between impaired glucose metabolism and reduced skeletal muscle mass; in diabetic populations, the prevalence of sarcopenia has been reported to reach 18%, which is markedly higher than in nondiabetic individuals.^[13]^ Notably, skeletal muscle accounts for approximately 80% of glucose uptake in the human body and serves as a key effector organ for maintaining glucose homeostasis. Once skeletal muscle mass declines or muscle function becomes impaired, insulin-mediated glucose disposal capacity is correspondingly reduced, leading excess glucose to be ectopically deposited as fat, thereby further aggravating metabolic dysregulation.^[14]^

Furthermore, protein metabolism plays a crucial role in maintaining skeletal muscle mass.^[15]^ Low serum albumin (ALB) levels have been shown to be significantly associated with sarcopenia, particularly among individuals with malnutrition or chronic diseases.^[16]^ Blood urea nitrogen (BUN), as a byproduct of nitrogen metabolism, is also regarded as an important indicator reflecting alterations in protein metabolic status.^[17]^ Body mass index (BMI), as a commonly used indicator of adiposity, may influence skeletal muscle mass through obesity-related metabolic abnormalities, whereas appendicular skeletal muscle index (ASMI), as a key index of skeletal muscle mass, can more accurately capture this association. Accordingly, dysregulation of lipid, glucose, and protein metabolism may play an important mediating role in the relationship between BMI and ASMI.

Moreover, obesity-related metabolic abnormalities may further affect skeletal muscle mass by inducing chronic inflammation, suggesting that inflammation may serve as an important mediating mechanism within this pathway.^[18]^ C-reactive protein (CRP), a sensitive indicator reflecting systemic inflammatory levels, is closely associated with the decline in skeletal muscle mass.^[19]^ A large-scale study involving 237,000 Korean adults demonstrated that elevated high-sensitivity CRP (hs-CRP) significantly increased the risk of sarcopenic obesity, with the association being more pronounced among women and individuals under 60 years of age.^[20]^ The systemic immune-inflammation index (SII), which integrates neutrophil, lymphocyte, and platelet counts, serves as a comprehensive indicator of immune-inflammatory status. In recent years, SII has been increasingly applied in studies of metabolic disorders and sarcopenia. A systematic review and meta-analysis conducted by Xie et al,^[21]^ encompassing 9 studies with a total of 18,634 adults, demonstrated that elevated SII levels were significantly associated with an increased risk of sarcopenia, with high-SII individuals exhibiting approximately 52% higher risk (OR = 1.52, 95% CI: 1.09–2.13, *P* = .01). Taken together, chronic low-grade inflammation, a common pathophysiological feature of obesity, is closely associated with the decline in skeletal muscle mass and may act as an important mediator in the relationship between BMI and ASMI.^[22,23]^

BMI may also directly influence skeletal muscle mass. A high BMI may increase absolute muscle mass through mechanical loading effects, but may also impair muscle quality due to greater intramuscular fat infiltration.^[24]^ In contrast, a low BMI may reduce joint loading but increase the risk of disuse-related muscle atrophy.^[25]^ Therefore, the relationship between elevated BMI and skeletal muscle mass may involve not only direct effects but also complex indirect effects mediated through metabolic and inflammatory mechanisms.^[26,27]^ However, previous studies have mainly focused on isolated mechanisms or associations between individual variables and have lacked an integrated analytical framework incorporating both metabolic and inflammatory markers to examine their direct and indirect effects in the relationship between BMI and ASMI. Accordingly, the present study aimed to: elucidate the relationships between ASMI and BMI, glucose, lipid, protein, inflammatory, and other related variables; identify independent risk and protective factors associated with reduced skeletal muscle mass; and investigate the mediating pathways and effect magnitudes of metabolic and inflammatory mechanisms in the association between BMI and ASMI. Through these analyses, this study may contribute to a better understanding of factors associated with skeletal muscle mass decline in non-elderly adults, while also providing potential evidence for more targeted risk assessment and early prevention strategies for sarcopenia.

## 2. Methods

Study data were collected from outpatients undergoing routine health examinations at a provincial hospital in Taiyuan, China between June 2019 and January 2023. Most participants were local residents. All participants provided written informed consent. The study followed the Declaration of Helsinki and was approved by the Ethics Committee of Shanxi University (IRB No. SXULL2022047).

Adults aged 18 to 59 years were included in this study. Data collected included anthropometric measurements, blood test results, and questionnaire survey data. Anthropometric measurements included height, weight (with BMI calculated from measured data), and appendicular skeletal muscle mass of the upper and lower limbs assessed using dual-energy X-ray absorptiometry. In addition, lifestyle-related information, including physical activity, smoking, and alcohol consumption, was obtained through questionnaires. To reduce potential measurement bias, all laboratory measurements were conducted using standardized procedures. Blood samples collected during the health examination were analyzed using standardized automated biochemical analyzers. Enzymatic methods were used to measure serum TC, TG, LDL-C, HDL-C, ALB, and BUN levels. HbA1c was measured using high-performance liquid chromatography, while INS levels were determined using chemiluminescent immunoassay. CRP levels were measured using immunoturbidimetry. SII was calculated from platelet, neutrophil, and lymphocyte counts obtained from routine blood tests, as shown in Equation (1). Participants with missing data on variables of interest, pregnancy at the time of the survey, or a history of cancer or type 1 diabetes were excluded. After applying these criteria, a total of 2277 participants were retained for the final analysis (1092 men and 1185 women).

Blood samples collected during the health examination were used to obtain laboratory indicators. Lipid indicators included TC, TG, LDL-C, and HDL-C. Glucose metabolism indicators included HbA1c and INS. Protein metabolism indicators included ALB and BUN. Inflammatory indicators included CRP and SII.


$$SII=\frac{Platelet\times Neutrophil}{Lymphocyte}$$ (1)

BMI was calculated using height and weight measured during the health examination and categorized according to the World Health Organization classification: BMI ≥ 30 kg/m^2^ was defined as obese and BMI < 30 kg/m^2^ as nonobese. Appendicular lean mass was obtained from dual-energy X-ray absorptiometry measurements, and the ASMI was calculated as appendicular lean mass divided by height squared (kg/m^2^).


$$\begin{array}{c} ASMI=\frac{Appendicular\;Lean\;Mass}{Height^{2}}\left(kg/m^{2}\right) \end{array}$$ (2)

Lifestyle information was obtained from questionnaire surveys conducted during the health examination, including physical activity, smoking status, and alcohol consumption. According to the Physical Activity Guidelines,^[28]^ participants were classified into low (< 500 metabolic equivalents of task [MET] min/week) and high (≥ 500 MET min/week) physical activity groups. Smoking status was categorized as never or former/current smoker. Alcohol consumption was classified as never, moderate (< 2 times per week), or heavy (≥ 2 times per week).

Participant characteristics are presented in Table 1. Continuous variables are expressed as mean ± SD and categorical variables as n (%). Spearman correlation analysis examined relationships between ASMI and continuous variables. Random forest was used to assess variable importance. Linear regression evaluated associations between predictors and ASMI, while logistic regression identified factors associated with reduced muscle mass. Piecewise structural equation modeling (SEM) was applied to examine direct and indirect effects of BMI, metabolic, and inflammatory indicators on ASMI while accounting for random effects of sex and physical activity. Variables were standardized prior to SEM analysis.

**Table 1:** General demographic characteristics.

Variables	Total (n = 2277)	Obesity		Statistic	*P*-value
		0 (n = 1456)	1 (n = 821)		
AGE, Mean ± SD	38.26 ± 12.51	37.40 ± 12.73	39.79 ± 11.97	*t* = −4.48	**<.001**
Weight, Mean ± SD	79.99 ± 20.50	69.06 ± 12.14	99.37 ± 17.80	*t* = −43.43	**<.001**
Height, Mean ± SD	166.84 ± 9.36	167.35 ± 9.45	165.93 ± 9.11	*t* = 3.51	**<.001**
BMI, Mean ± SD	28.69 ± 6.84	24.57 ± 3.24	36.00 ± 5.27	*t* = −56.43	**<.001**
ASM index, Mean ± SD	7.96 ± 1.70	7.27 ± 1.37	9.18 ± 1.54	*t* = −30.50	**<.001**
HDL C, Mean ± SD	1.38 ± 0.39	1.45 ± 0.41	1.25 ± 0.33	*t* = 12.98	**<.001**
TG, Mean ± SD	1.17 ± 0.75	1.08 ± 0.72	1.34 ± 0.77	*t* = −8.01	**<.001**
LDL C, Mean ± SD	2.91 ± 0.88	2.86 ± 0.87	3.00 ± 0.88	*t* = −3.83	**<.001**
TC, Mean ± SD	4.83 ± 1.00	4.80 ± 0.99	4.87 ± 1.01	*t* = −1.49	.136
SII, Mean ± SD	473.71 ± 267.70	445.19 ± 249.62	524.28 ± 290.42	*t* = −6.56	**<.001**
HbA1c, Mean ± SD	5.64 ± 1.08	5.48 ± 0.86	5.92 ± 1.35	*t* = −8.51	**<.001**
CRP, Mean ± SD	3.80 ± 8.30	2.41 ± 6.52	6.28 ± 10.31	*t* = −9.72	**<.001**
INS, Mean ± SD	75.26 ± 80.03	51.08 ± 35.98	118.15 ± 112.26	*t* = −16.65	**<.001**
Serum Albumin, Mean ± SD	42.21 ± 3.61	42.97 ± 3.42	40.87 ± 3.55	*t* = 13.90	**<.001**
BUN, Mean ± SD	4.73 ± 1.57	4.72 ± 1.50	4.74 ± 1.70	*t* = −0.33	.739
SEX, n(%)				χ^2^ = 19.64	**<.001**
Male	1092 (47.96)	749 (51.44)	343 (41.78)		
Female	1185 (52.04)	707 (48.56)	478 (58.22)		
Education, n(%)				*χ*^2^ = 3.77	.152
Less than high school	453 (19.89)	273 (18.75)	180 (21.92)		
High school or equivalent	553 (24.29)	365 (25.07)	188 (22.90)		
College or above	1271 (55.82)	818 (56.18)	453 (55.18)		
Smoking, n(%)				*χ*^2^ = 2.34	.126
No	1433 (62.96)	933 (64.12)	500 (60.90)		
Yes	843 (37.04)	522 (35.88)	321 (39.10)		
Drinking, n(%)				*χ*^2^ = 2.81	.245
No	702 (30.83)	439 (30.15)	263 (32.03)		
Moderate drinking	1498 (65.79)	973 (66.83)	525 (63.95)		
Heavy drinking	77 (3.38)	44 (3.02)	33 (4.02)		
Mets, n(%)				*χ*^2^ = 0.24	.624
Low	1177 (51.69)	747 (51.30)	430 (52.38)		
High	1100 (48.31)	709 (48.70)	391 (47.62)		

Data are presented as mean ± SD for continuous variables and n (%) for categorical variables. Differences between groups were assessed using the independent-samples *t* test for continuous variables and the *χ*^2^ test for categorical variables. Bold values indicate statistical significance (*P* < .05).

ASMindex = appendicular skeletal muscle mass index, BMI = body mass index, BUN = blood urea nitrogen, CRP = C-reactive protein, HbA1c = glycated hemoglobin, HDL-C = high-density lipoprotein cholesterol, INS = insulin, LDL-C = low-density lipoprotein cholesterol, METs = metabolic equivalents of task, SD = standard deviation, SII = systemic immune-inflammation index, TC = total cholesterol, TG = triglycerides.

Statistical analyses and visualization were performed in R (version 4.5.0). Random forest, regression models, and piecewise SEM were implemented using standard R packages, with significance defined as *P* < .05. Model fit in SEM was evaluated using Fisher C test (.05 < *P* < 1.00).

## 3. Results

### 3.1. Descriptive statistics

Significant differences were observed between obese and nonobese participants across most continuous variables (Table 1). The obese group had higher age, body weight, BMI, and ASMI (all *P* < .001). Sex distribution also differed between groups, with a higher proportion of females in the obese group (*P* < .001), whereas no significant differences were observed in education, smoking, alcohol consumption, or physical activity (METs). Regarding metabolic and inflammatory indicators, ALB levels were significantly lower in the obese group (*P* < .001), whereas BUN showed no significant difference between groups (*P* = .739). Levels of HDL-C were significantly decreased among obese participants, while TG, LDL-C, HbA1c, and INS levels were all significantly elevated (*P* < .001). In addition, inflammatory markers CRP and SII were both significantly higher in the obese group compared with the nonobese group (*P* < .001).

### 3.2. Variable importance analysis by random forest

**Figure 1.**
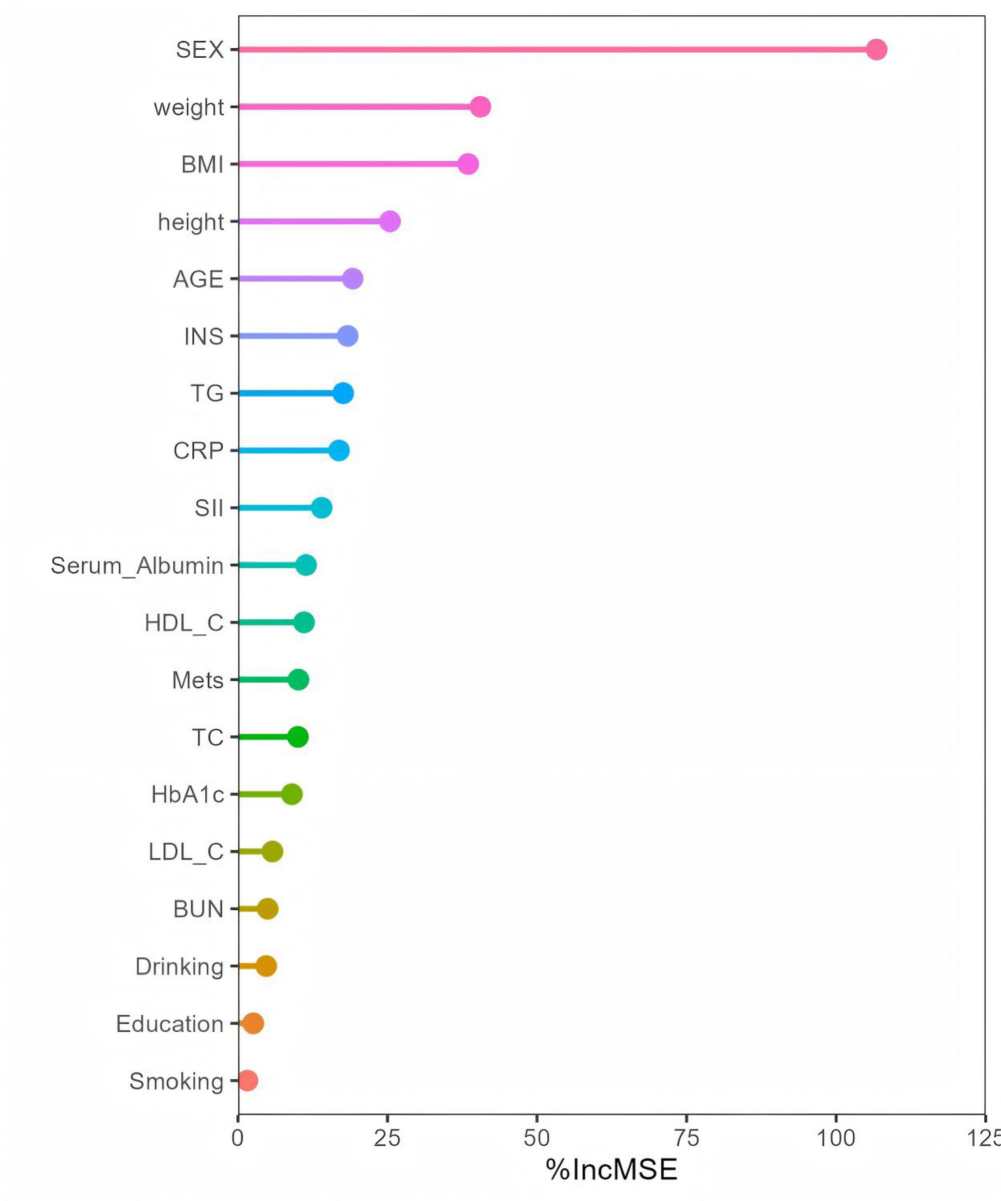
Random forest variable importance analysis. Variable importance was assessed using the percentage increase in mean squared error (%IncMSE), with higher values indicating greater. BMI = body mass index, BUN = blood urea nitrogen, CRP = C-reactive protein, HbA1c = glycated hemoglobin, HDL-C = high-density lipoprotein cholesterol, INS = insulin, LDL-C = low-density lipoprotein cholesterol, METs = metabolic equivalents of task, SII = systemic immune-inflammation index, TC = total cholesterol, TG = triglycerides.

Figure 1 illustrates the variable importance ranking in the random forest model, with ASMI set as the dependent variable. The results indicated that sex, body weight, and BMI were the top 3 predictors contributing most substantially to ASMI prediction. Among these, sex exhibited the highest importance, markedly exceeding that of all other variables.

Variables such as height, INS, TG, CRP, AGE, HDL-C, SII, ALB, and TC also demonstrated notable influence on ASMI prediction. In contrast, BUN and lifestyle-related variables such as smoking contributed relatively little to the model’s predictive performance.

### 3.3. Correlation analysis

**Figure 2.**
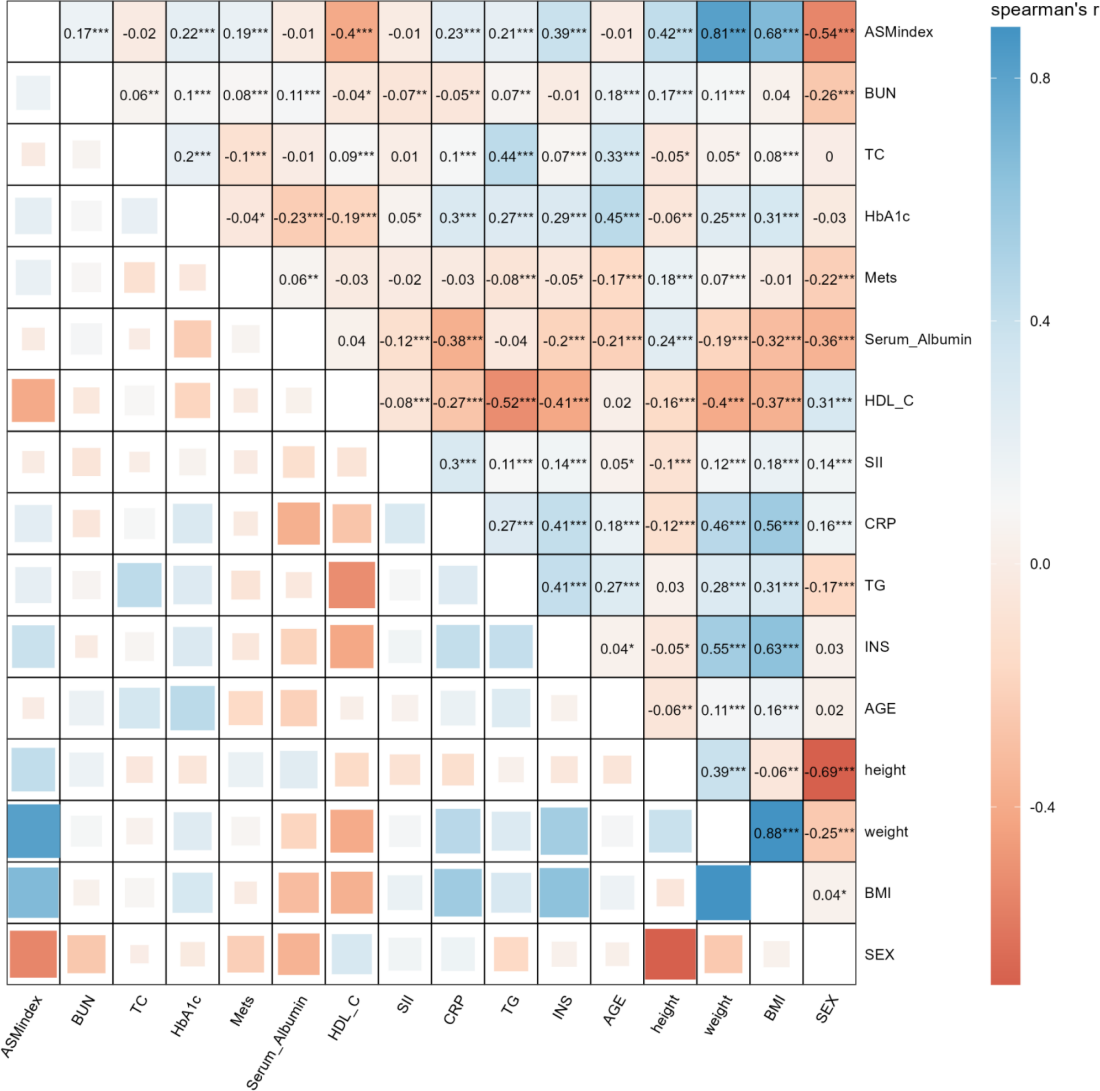
Spearman correlation analysis among metabolic indicators, inflammatory factors, ASMI, BMI, and other continuous variables. The values represent correlation coefficients, and asterisks indicate significance levels (*P < .05, **P < .01, and ***P < .001). BMI = body mass index, BUN = blood urea nitrogen, CRP = C-reactive protein, HbA1c = glycated hemoglobin, HDL-C = high-density lipoprotein cholesterol, INS = insulin, LDL-C = low-density lipoprotein cholesterol, METs = metabolic equivalents of task, SII = systemic immune-inflammation index, TC = total cholesterol, TG = triglycerides.

Spearman correlation analysis was performed on 15 numeric variables identified by the random forest method (Fig. 2). ASMI was positively correlated with BMI (*r* = 0.68), BUN (*r* = 0.17), HbA1c (*r* = 0.22), CRP (*r* = 0.23), TG (*r* = 0.21), and INS (*r* = 0.39), and negatively correlated with HDL-C (*r* = −0.40) and sex (*r* = −0.54; all *P* < .001). BMI was positively correlated with inflammatory and glucose metabolic indicators and negatively correlated with HDL-C (*r* = −0.37) and ALB (*r* = −0.32; all *P* < .001). Additional correlations of varying magnitudes were observed among the remaining variables.

### 3.4. Multiple linear regression analysis

A multiple linear regression model was constructed using the 15 variables identified by the random forest method to examine factors associated with ASMI (Table 2), showing good model fit (adjusted *R*^2^ = 0.833). BMI (*β* = 0.09, 95% CI: 0.05–0.13, *P* < .001), HDL-C (*β* = 0.25, 95% CI: 0.15–0.30, *P* < .001), and LDL-C (*β* = 0.19, 95% CI: 0.08–0.30, *P* < .001) were positively associated with ASMI, whereas CRP (*β* = –0.01, 95% CI: −0.99 to −0.01, *P* < .05), SII (*β* = −0.01, 95% CI: −0.99 to −0.01, *P* < .001), and TC (*β* = −0.20, 95% CI: −0.30 to −0.10, *P* < .05) were negatively associated. Body weight and higher physical activity were positively associated with ASMI, while height, age, and female sex showed negative associations.

**Table 2:** Linear regression analysis of factors influencing skeletal muscle mass.

Variables	*β*	S.E	*t*	*P*	*β* (95% CI)
Weight	0.04	0.01	5.22	<.001	0.04 (0.02 to 0.05)
Height	−0.02	0.01	−2.33	.020	−0.02 (−0.03 to −0.01)
BMI	0.09	0.02	4.56	<.001	0.09 (0.05 to 0.13)
CRP	−0.01	0.00	−2.46	.014	−0.01 (−0.01 to −0.01)
SII	−0.01	0.00	−5.88	<.001	−0.01 (−0.01 to −0.01)
HDL-C	0.25	0.05	4.69	<.001	0.25 (0.15 to 0.36)
LDL-C	0.19	0.06	3.32	<.001	0.19 (0.08 to 0.30)
TC	−0.20	0.05	−3.91	<.001	−0.20 (−0.30 to −0.10)
Age	−0.01	0.00	−8.28	<.001	−0.01 (−0.01 to −0.01)
Sex					
Male					0.00 (Reference)
Female	−1.67	0.04	−39.02	<.001	−1.67 (−1.76 to −1.59)
Mets (graded)					
Low					0.00 (Reference)
High	0.17	0.03	5.67	<.001	0.17 (0.11 to 0.23)

BMI = body mass index, CI = confidence interval, CRP = C-reactive protein, HDL-C = high-density lipoprotein cholesterol, LDL-C = low-density lipoprotein cholesterol, METs = metabolic equivalents of task, S.E = standard error, SII = systemic immune-inflammation index, TC = total cholesterol, *β* = regression coefficient.

### 3.5. Multiple logistic regression analysis

**Figure 3.**
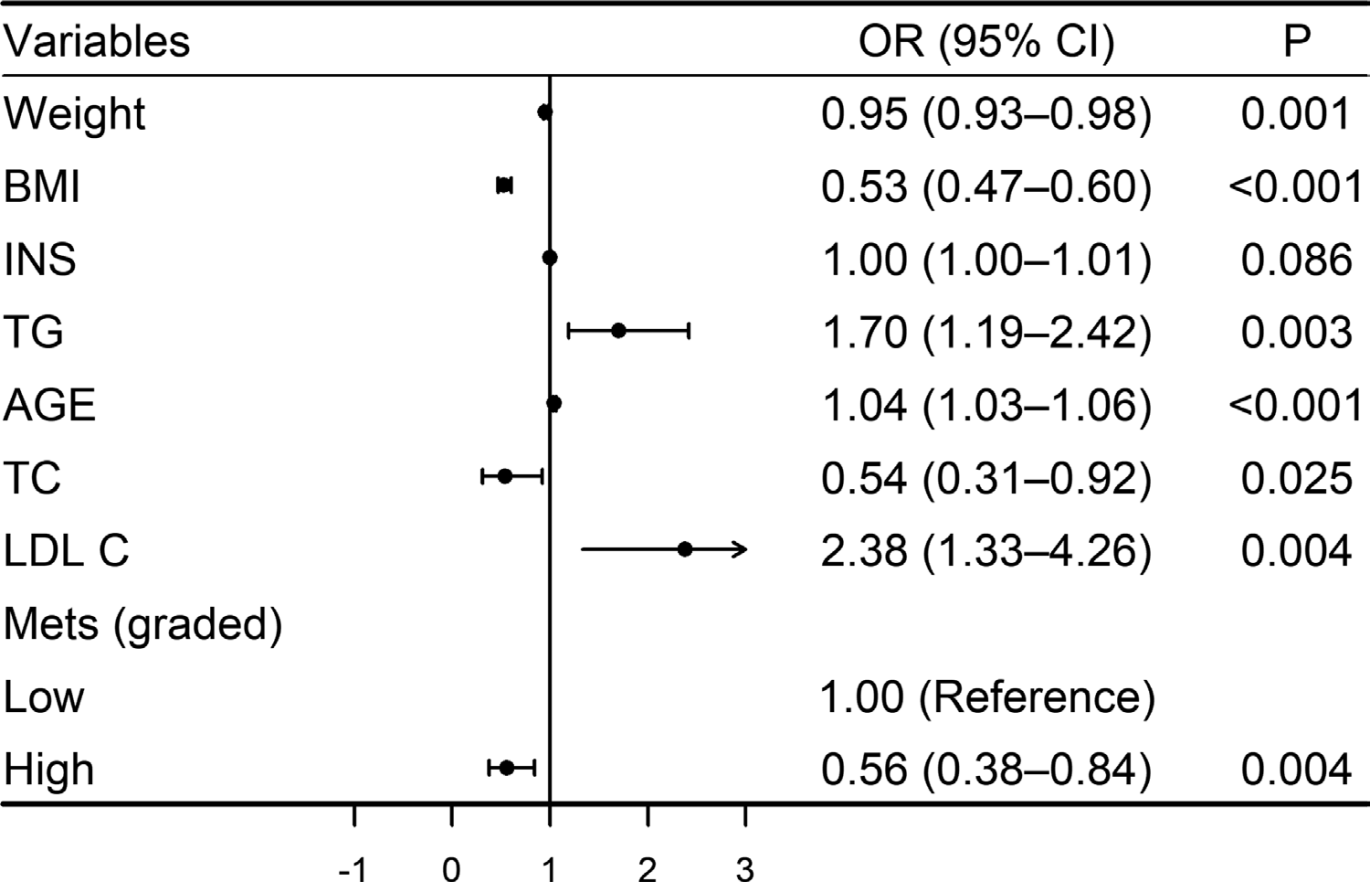
Logistic regression analysis of factors influencing skeletal muscle mass. Odds ratios (ORs) and 95% confidence intervals (CIs) are presented. The vertical line represents OR = 1. Variables with OR > 1 indicate increased risk, whereas OR < 1 indicate decreased risk. Low physical activity was used as the reference category for metabolic equivalent tasks (METs). BMI = body mass index, INS = insulin, LDL-C = low-density lipoprotein cholesterol, METs = metabolic equivalents of task, TC = total cholesterol, TG = triglycerides.

ASMI was dichotomized into low and normal groups according to sex-specific sarcopenia thresholds (< 7.0 kg/m^2^ in men; < 5.4 kg/m^2^ in women).^[29]^ Using low ASMI as the outcome, 15 variables identified by random forest were entered into a bidirectional stepwise multivariable logistic regression model (Fig. 3). Higher BMI was associated with a lower risk of low skeletal muscle mass (OR = 0.53, 95% CI: 0.47–0.60, *P* < .001). Body weight and physical activity (METs) were also inversely associated with risk (weight OR = 0.95, *P* = .001; METs OR = 0.56, *P* = .004), whereas older age was associated with increased risk (OR = 1.04, 95% CI: 1.03–1.06, *P* < .001). Among metabolic indicators, higher TG (OR = 1.70, *P* = .003) and LDL-C (OR = 2.38, *P* = .004) were positively associated with risk, while TC showed an inverse association (OR = 0.54, *P *< .05). INS was not significantly associated (*P* = .086).

### 3.6. SEM analysis

**Figure 4.**
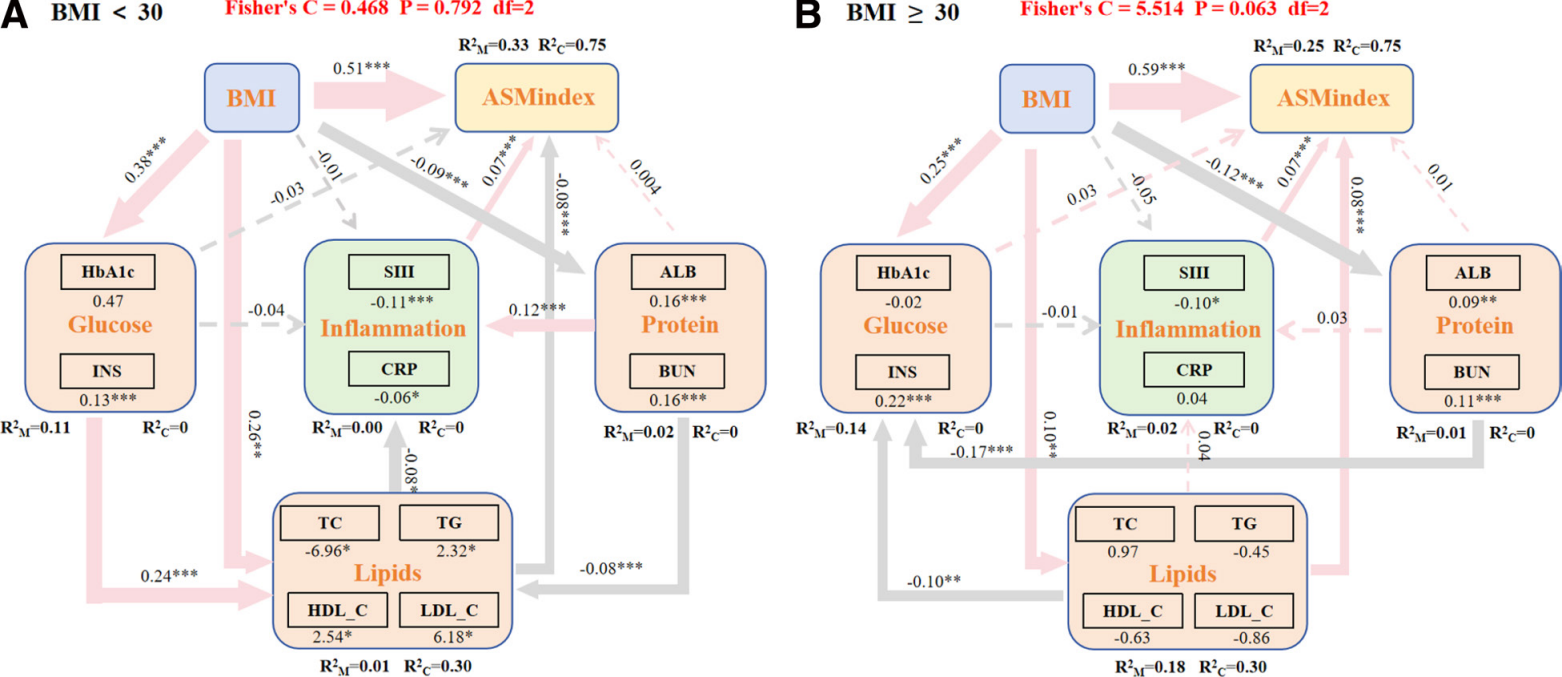
Piecewise structural equation model revealing the BMI–ASMI relationship and mediation pathway. (A) represents participants with BMI < 30 (non-obese group), and (B) represents participants with BMI ≥ 30 (obese group). The variables representing glucose, lipid, protein, and inflammation were each combined into composite variables. The numbers shown beside each variable name indicate their coefficients with respect to the standardized composite variable. The numbers along the arrows represent path coefficients, reflecting the magnitude of the direct standardized effects among these relationships. The thickness of the arrows corresponds to the strength of the relationships, with solid arrows indicating statistically significant paths and dashed arrows representing nonsignificant paths. The total standardized effects of BMI and the composite variables on ASMI are displayed as marginal *R*^2^ and conditional *R*^2^. The significance levels for each predictor are denoted as *P < .05, **P < .01, and ***P < .001. ALB = albumin, BMI = body mass index, BUN = blood urea nitrogen, CRP = C-reactive protein, HbA1c = glycated hemoglobin, HDL-C = high-density lipoprotein cholesterol, INS = insulin, LDL-C = low-density lipoprotein cholesterol, SII = systemic immune-inflammation index, TC = total cholesterol, TG = triglycerides.

A piecewise SEM was used to examine the direct and indirect effects of BMI, glucose-related, lipid-related, protein-related, and inflammatory indicators on ASMI. As shown in Figure 4, BMI exerted indirect effects on ASMI through multiple metabolic and inflammatory pathways, with pathway strength and direction varying across BMI levels. The direct path from BMI to ASMI was retained in both models. Considering random effects in the ASMI model, BMI together with metabolic and inflammatory factors explained a substantial proportion of ASMI variation in both nonobese (BMI < 30, 75%) and obese (BMI ≥ 30, 75%) participants.

In the nonobese group (BMI < 30), the model showed good fit (Fisher C = 0.468, *P* = .792, df = 2). Inflammation had a significant positive effect on ASMI (*β* = 0.07, *P* < .001). Lipids showed a negative direct effect on ASMI (*β* = −0.08, *P* < .001) and an indirect effect through their negative association with inflammation (*β* = −0.08, *P* < .05). Proteins also showed an indirect pathway to ASMI via a positive effect on inflammation (*β* = 0.12, *P* < .001). Interrelationships were observed among metabolic modules: glucose positively regulated lipids (*β* = 0.24, *P* < .001), whereas proteins were negatively associated with lipids (*β* = −0.08, *P* < .001).

In the obese group (BMI ≥ 30), the model also showed acceptable fit (Fisher C = 5.514, *P* = .063, df = 2). BMI showed positive effects on glucose (*β* = 0.25, *P* < .001) and lipids (*β* = 0.10, *P* < .001) and a negative effect on proteins (*β* = −0.12, *P* < .001), whereas the paths from metabolic modules to inflammation were not significant. Compared with the nonobese group, lipids showed a positive effect on ASMI (*β* = 0.08, *P* < .001), while inflammation remained significantly associated with ASMI (*β* = 0.07, *P* < .001). In addition, both lipids and proteins showed negative effects on glucose.

## 4. Discussion

The reduction of skeletal muscle mass has been recognized by the WHO and several other international health authorities as a major public health challenge for the future.^[30]^ Previous studies have shown that decreased skeletal muscle mass often coexists with multiple chronic conditions such as osteoporosis, obesity, metabolic syndrome, and type 2 diabetes mellitus, forming complex bidirectional relationships that exacerbate functional decline and increase disease burden.^[31,32]^ Moreover, metabolic abnormalities and chronic inflammation may play important mediating roles in skeletal muscle mass reduction, serving as key physiological mechanisms linking body composition and skeletal muscle mass.^[33]^

In both the correlation and multiple linear regression analyses of this study, BMI showed a significant positive association with ASMI, consistent with previous findings.^[34]^ This relationship may be explained by the fact that muscle and fat together account for a substantial proportion of total body mass; therefore, without precise body composition differentiation, increases in BMI among non-elderly individuals generally reflect simultaneous gains in both fat and skeletal muscle mass, particularly in those with higher levels of physical activity.^[35]^ Furthermore, the logistic regression results indicated that higher BMI significantly reduced the risk of low skeletal muscle mass. Yang et al^[36]^ also reported that BMI served as an independent protective factor for ASMI among healthy Chinese adult males. Similarly, another study involving 926 individuals without sarcopenia found that higher BMI had a protective effect on skeletal muscle mass in both men (OR = 0.344, 95% CI: 0.213–0.555, *P* < .001) and women (OR = 0.289, 95% CI: 0.174–0.480, *P* < .001).^[37]^

Among the metabolic indicators, lipid parameters showed particularly strong associations with skeletal muscle mass. In this study, HDL-C was positively correlated with ASMI, consistent with its established physiological roles in maintaining metabolic homeostasis through anti-inflammatory, antioxidative, and reverse cholesterol transport mechanisms.^[38]^ In the present study, TC appeared as a protective factor. Previous research has suggested that moderately elevated TC levels may reflect adequate nutritional reserves and energy availability, thereby contributing to the maintenance of skeletal muscle mass.^[39]^ In contrast, elevated triglyceride (TG) and LDL-C levels were considered risk factors for reduced skeletal muscle mass, consistent with previous studies in Asian populations reporting elevated TG levels as a risk factor for sarcopenia.^[40]^ The underlying mechanism may involve excessive lipid accumulation inducing ectopic fat deposition in skeletal muscle, thereby impairing muscle anabolic metabolism through oxidative stress and inflammatory responses.^[41]^ Moreover, elevated LDL-C levels, particularly under conditions of insulin resistance or chronic inflammation, may further aggravate vascular injury and inflammatory responses, ultimately contributing to skeletal muscle mass decline.^[42]^ Notably, skeletal muscle serves as a major site for peripheral fatty acid oxidation, and reductions in skeletal muscle mass may directly impair mitochondrial oxidative capacity, hinder lipid clearance, and induce metabolic imbalance, resulting in elevated TG levels and reduced HDL-C levels, thereby further aggravating lipid dysregulation.^[43]^ Regarding glucose metabolism, insulin resistance may suppress the PI3K/Akt/mTOR signaling pathway, thereby impairing muscle protein synthesis and promoting protein catabolism, ultimately accelerating skeletal muscle mass decline.^[44]^ Conversely, reductions in skeletal muscle mass may also decrease GLUT4-mediated glucose uptake, impair peripheral insulin sensitivity, and potentially form a vicious cycle through reciprocal interactions.^[45]^ In the present study, HbA1c, INS, ALB, and BUN did not show significant direct associations with ASMI, which may be related to the relatively young study population, generally favorable metabolic status, and preserved self-regulatory capacity.

Inflammation was also found to have a substantial impact on skeletal muscle mass. The present results indicated that for every 1 mg/L increase in CRP, ASMI decreased by an average of 0.01 kg/m^2^, and for every 1 × 10^9^/L increase in SII, ASMI decreased by 0.01 kg/m^2^, consistent with previous findings.^[46]^ Choi et al reported that elevated levels of hs-CRP were independently associated with low skeletal muscle mass among Korean adult males.^[47]^ CRP may suppress muscle protein synthesis and promote the release of pro-inflammatory cytokines, thereby accelerating muscle loss. As a marker reflecting systemic inflammatory status, SII has also been shown to be independently associated with reduced skeletal muscle mass (*β* = 1.002, 95% CI: 1.001–1.003, *P* < .001), with ROC analysis identifying an optimal SII threshold of > 765 for predicting sarcopenia.^[48]^ Elevated SII typically reflects an increase in neutrophils and platelets and a reduction in lymphocytes, forming an inflammatory environment that may promote muscle protein degradation and skeletal muscle loss through mechanisms similar to those mediated by CRP.^[49]^

However, the specific mediating mechanisms linking BMI and ASMI remain incompletely understood. According to the SEM results of this study, inflammation significantly mediated the relationship between BMI and ASMI across BMI groups. Lipids exhibited different patterns between BMI categories: in the nonobese group, lipids exerted both direct effects on ASMI and indirect effects through inflammation, forming a “lipid–inflammation–ASMI” pathway; whereas in the obese group, the direct effect of lipids on ASMI was strengthened while the indirect mediation through inflammation was attenuated. This difference may indicate that lipid metabolic disorders exert stronger direct influences on skeletal muscle mass under obese conditions, whereas in nonobese individuals lipid abnormalities may affect muscle mainly through inflammatory pathways. Previous studies have shown that lipid metabolism disorders may lead to incomplete fatty acid oxidation, increased reactive oxygen species (ROS) production, and activation of oxidative stress and inflammatory signaling pathways, thereby impairing insulin sensitivity and suppressing muscle protein synthesis.^[50]^ In addition, ectopic lipid deposition-induced myosteatosis and mitochondrial dysfunction may further promote ROS accumulation, synergistically aggravating these metabolic disturbances and ultimately contributing to skeletal muscle mass decline.^[51]^ Slavin et al^[52]^ reported that ROS accumulation could activate the NLRP3 inflammasome and its downstream cascade reactions, thereby establishing a pro-inflammatory microenvironment within myocytes. Rubio-Ruiz et al^[53]^ further demonstrated that immune cell infiltration in hypertrophic adipose tissue may amplify systemic inflammatory signaling. A clinical study by Guo et al^[54]^ showed that the hs-CRP/HDL-C ratio was positively associated with sarcopenia risk, with each unit increase corresponding to a 6% increase in sarcopenia risk (OR = 1.06, 95% CI: 1.04–1.08, *P* = .004), suggesting synergistic effects of lipid dysregulation and inflammatory pathways on skeletal muscle loss. Lin et al^[55]^ further reported an inverted J-shaped association between CRP levels and the risk of low skeletal muscle mass, with an inflection point at 0.273 mg/dL, indicating that the influence of inflammation on skeletal muscle may be dose-dependent and may exert significant negative effects only after exceeding a certain threshold. Consistent with these findings, the SEM results of the present study showed a positive association between mild inflammation and ASMI in a predominantly non-elderly population, corresponding to the low-dose range of this nonlinear association.

Although glucose and protein metabolism did not show significant mediating effects in the SEM, they may still indirectly influence ASMI through interactions with lipid metabolism and inflammatory pathways. Notably, BMI was directly associated with ASMI and showed significant associations with glucose, lipid, and protein variables, whereas its direct effect on inflammation was not significant. This suggests that BMI may influence inflammation indirectly through metabolic disturbances. Previous studies have reported strong associations between elevated BMI and metabolic abnormalities.^[56,57]^ Meanwhile, chronic low-grade inflammation has been recognized as an important contributor to skeletal muscle decline under conditions of metabolic dysfunction. Buchmann et al^[58]^ reported significant associations between inflammation and skeletal muscle mass, noting that elevated CRP and IL-6 levels accelerate muscle loss. Similarly, Liu et al^[59]^ emphasized that the development of sarcopenia involves interactions among metabolic, inflammatory, and immune systems. Conversely, reductions in skeletal muscle mass may themselves further aggravate chronic low-grade inflammation. Skeletal muscle possesses both metabolic and endocrine functions and can secrete anti-inflammatory myokines such as irisin and IL-15.^[60]^ Loss of skeletal muscle mass may weaken these anti-inflammatory regulatory effects, thereby contributing to the persistence of a pro-inflammatory state and interacting with inflammation-driven muscle protein catabolism, ultimately accelerating skeletal muscle mass decline.^[61]^

This study revealed distinct association patterns between lipid and inflammatory factors and skeletal muscle mass across different BMI categories in a predominantly non-elderly population, suggesting that sarcopenia may involve the combined effects of multiple metabolic and inflammatory mechanisms and further highlighting the importance of BMI stratification in skeletal muscle health risk assessment. Notably, even among individuals with normal BMI, lipid abnormalities may still significantly affect skeletal muscle mass through chronic inflammatory pathways, suggesting that this population may also carry a non-negligible risk of impaired muscle health. Combined evaluation of lipid and inflammatory indicators may therefore help identify early risk signals in these individuals. Due to the cross-sectional design, causal relationships could not be established in this study. Furthermore, incomplete medication information may have introduced residual confounding, and further studies are needed to validate the present findings.

## 5. Conclusion

BMI was positively associated with skeletal muscle mass and inversely associated with the risk of low skeletal muscle mass among non-elderly adults. Skeletal muscle mass was also related to metabolic, inflammatory, anthropometric, and behavioral factors. Lipid metabolic indicators demonstrated bidirectional associations with skeletal muscle mass, with TG and LDL-C identified as risk factors and TC as a protective factor. Distinct patterns were observed across BMI groups: in obese individuals lipid metabolism was mainly directly associated with skeletal muscle mass, whereas in nonobese individuals lipid metabolism was additionally associated with skeletal muscle mass through inflammation. Glucose and protein metabolism were indirectly associated with skeletal muscle mass via lipid-related pathways. These findings may help identify individuals at risk of low skeletal muscle mass and inform targeted prevention strategies.

## Acknowledgments

The authors thank the staff involved in data collection and management for their support. We are also grateful to all participants who took part in the health examination program and made this study possible.

## Author contributions

**Conceptualization:** Jingran Hu, Fei Wang.

**Data curation:** Jingran Hu, Bin Gu.

**Formal analysis:** Jingran Hu, Fei Wang.

**Funding acquisition:** Fei Wang.

**Methodology:** Jingran Hu, Fei Wang.

**Project administration:** Bin Gu, Fei Wang.

**Resources:** Jingran Hu, Fei Wang.

**Software:** Jingran Hu.

**Supervision:** Fei Wang.

**Validation:** Jingran Hu, Fei Wang.

**Visualization:** Jingran Hu, Bin Gu, Fei Wang.

**Writing – original draft:** Jingran Hu.

**Writing – review & editing:** Bin Gu, Fei Wang.
